# Relationship between hs-CRP-triglyceride glucose index and coronary plaque morphology: insights from optical coherence tomography

**DOI:** 10.1186/s13098-026-02227-8

**Published:** 2026-07-09

**Authors:** Hongying Yao, Qianyun Guo, Qian Ma, Yujing Cheng, Xuezhi Chen, Yujie Zhou

**Affiliations:** 1 Department of Cardiology, Peking University International Hospital, Beijing, 102206 China; 2 Department of Cardiology, Beijing Anzhen Hospital, Beijing Institute of Heart Lung and Blood Vessel Disease, Beijing Key Laboratory of Precision Medicine of Coronary Atherosclerotic Disease, Clinical center for coronary heart disease, Capital Medical University, Beijing, 100029 China

**Keywords:** Atherosclerosis, Coronary artery disease, High sensitivity C-reactive protein (hs-CRP),, Triglyceride glucose(TyG) index, Healed plaque, Vulnerable plaque, Optical coherence tomography (OCT)

## Abstract

**Background:**

Accumulating evidence has underscored that coronary plaque morphology is the key determinant of acute cardiovascular events. The high-sensitivity C-reactive protein-triglyceride-glucose index (CTI) has been shown to correlate strongly with cardiovascular events and mortality. However, no previous study has investigated the relationship between CTI and detailed plaque morphological features evaluated using in vivo optical coherence tomography (OCT).

**Methods:**

In this retrospective cross-sectional study, 444 patients with coronary artery disease (CAD) who underwent OCT prior to percutaneous coronary intervention (PCI) were analyzed. The association between CTI and plaque morphology was examined employing multivariable logistic regression models. Stratified analyses according to clinical presentation (stable angina pectoris [SAP] vs. acute coronary syndrome [ACS]) and tests for interaction were further performed. Receiver operating characteristic (ROC) curve analysis was used to assess the discriminative ability of CTI for identifying vulnerable plaques.

**Results:**

The study revealed that participants divided into CTI quartiles exhibited a steadily rising prevalence of macrophage accumulation and healed plaque. Compared with the low CTI groups (Q1 and Q2), the incidences of cholesterol crystals and multiple healed plaques (≥ 2) were significantly higher in the high CTI groups (Q3 and Q4) (cholesterol crystals: 35.1% and 24.3% vs. 18.9% and 13.5%; multiple healed plaques: 18.9% vs. 12.6%). After accounting for the effects of different covariates, analyzing CTI as a continuous variable revealed a significant link to healed plaque (OR = 1.62, 95%CI:1.21–2.16, *P* = 0.001), cholesterol crystals (OR = 1.64, 95%CI:1.17–2.30, *P* = 0.004), macrophage accumulation (OR = 1.49, 95%CI:1.13–1.98, *P* = 0.005), and plaque rupture (OR = 1.54, 95%CI:1.07–2.21, *P* = 0.019). The statistically significant association persisted when CTI was split into quartiles. Notably, stratified analyses revealed that these significant associations were only present in patients with ACS. The RCS analysis demonstrated a linear association between CTI and various lipid plaque-related parameters. The area under the ROC curve (AUC) demonstrated that CTI exhibited a stronger correlation with plaque vulnerability compared with other single factors.

**Conclusion:**

In patients with ACS, elevated CTI is linked to an increased risk of healed plaque and vulnerable plaques. There is a linear relationship between CTI and various lipid plaque-related parameters. As a single factor, CTI provides a superior discriminative capacity for vulnerable plaque.

## Introduction

Coronary artery disease (CAD) remains a leading cause of mortality and morbidity worldwide, imposing a substantial burden on healthcare systems [1–3]. The progression of CAD is closely driven by the evolution of coronary atherosclerotic plaques, and accumulating evidence indicates that coronary plaque morphology, rather than just the severity of luminal stenosis, is the key determinant of acute cardiovascular events (such as myocardial infarction and sudden cardiac death) [4–7]. Specifically, vulnerable plaque features, such as thin-cap fibroatheromas (TCFAs), large lipid index, cholesterol crystals, macrophage accumulation, and active inflammation are strongly associated with plaque rupture, which triggers acute thrombotic complications [4]. Additionally, other morphological phenotype, such as healed plaque, which may present with vulnerable properties, or represent a stabilized phase following prior subclinical events, may perform a critical role in the development of arteriosclerosis [8–10].

Optical coherence tomography (OCT) has become the gold standard for in vivo assessment of coronary plaque morphology. With high-resolution imaging(10–20 μm), it enables quantitative measurement of fibrous cap thickness (FCT), lipid index, as well as identification of subtle features including lipid-rich plaque, macrophage accumulation, and healed plaques [4]. Using this sophisticated tool, researchers have explored the association between systemic biomarkers and plaque morphology to identify vulnerable or healed plaques [11–13].

Among these biomarkers, the triglyceride-glucose (TyG) index has gained attention as a simple and dependable indicator of insulin resistance [14, 15]. Elevated TyG levels are independently associated with an increased prevalence of vulnerable coronary plaques and coronary artery calcification [12, 16]. However, the TyG index primarily reflects metabolic dysregulation and fails to capture the inflammatory component of atherosclerosis, which is a critical driver of plaque instability [17–20]. Chronic low-grade inflammation is crucial at every stage of atherosclerotic plaque formation and development, from lipid accumulation to plaque rupture. High-sensitivity C-reactive protein (hs-CRP), an indicator of systemic inflammation, has been reliably associated with the risk of CAD, atherosclerotic plaque progression, and plaque vulnerability [21, 22]. Notably, inflammation and metabolism are closely correlated and may interact with each other, playing a crucial role in atherosclerosis [23].

Accordingly, a biomarker that integrates both dimensions may provide more comprehensive discriminative value than either alone. Recent studies have verified that CTI, a ‘metabolism-inflammation’ combined marker, is strongly correlated with cardiovascular events and mortality [24–27], though the underlying pathological mechanism remains unclear. This study was designed to address this gap by leveraging OCT’s high-resolution imaging capabilities, as the relationship between CTI and the microscopic characteristics of coronary plaques—especially vulnerable plaque features—has not been explored.

We hypothesized that elevated CTI would be associated with adverse coronary plaque morphological features, such as thinner FCT, larger lipid cores, and a higher prevalence of healed plaques. By clarifying this association, our findings could provide novel insights into the biological mechanisms that link metabolic-inflammatory dysregulation to plaque morphology and potentially identify a new biomarker for CAD risk stratification.

## Methods

### Study population and design

**Fig. 1:**
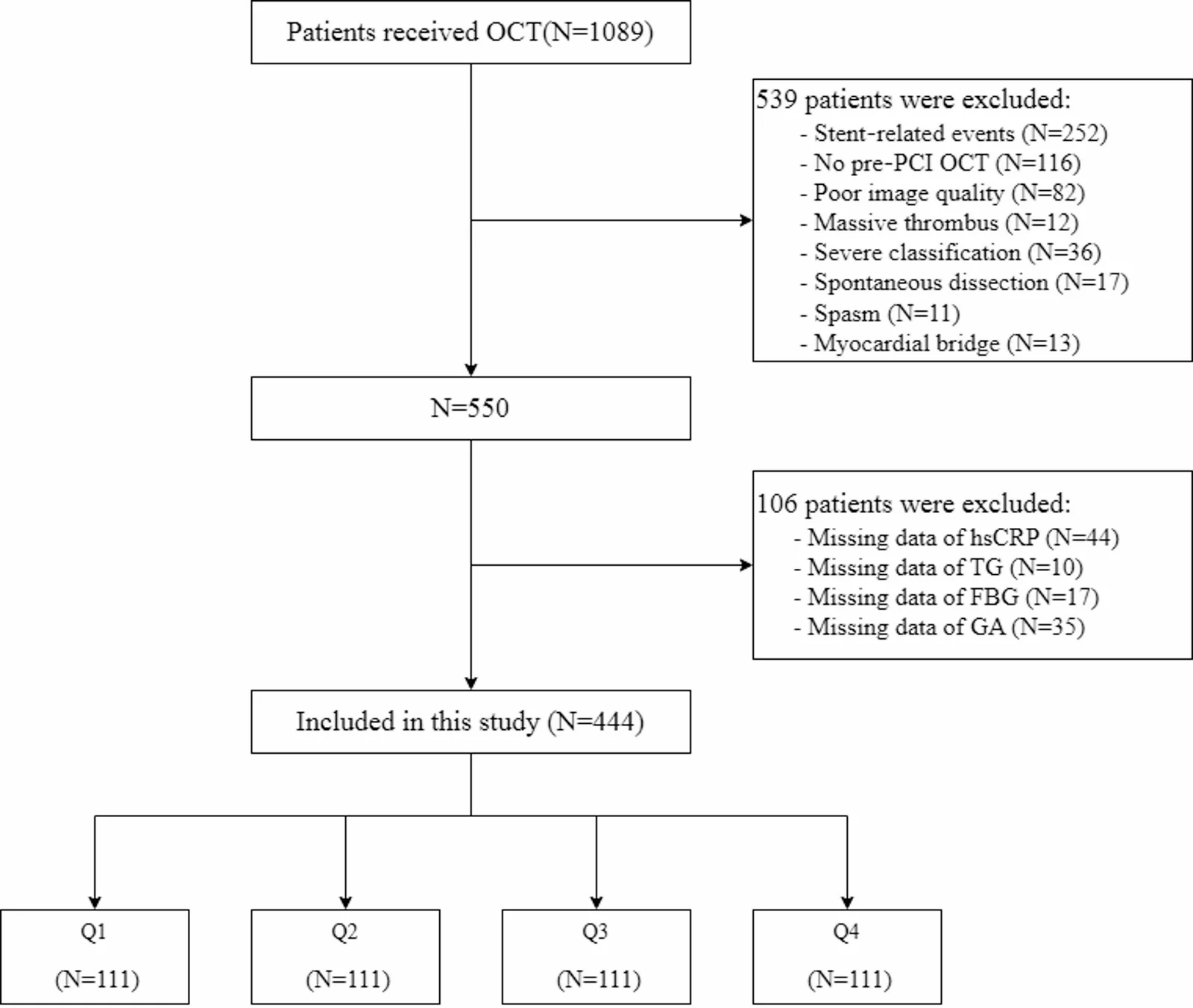
Flow chart of the study participants. OCT, optical coherence tomography; PCI, percutaneous coronary intervention; hsCRP, high-sensitivity C-reactive protein; TG, triglyceride; FBG, fasting blood glucose; GA, glycated albumin

A cross-sectional retrospective study was carried out at Ward 12 of Anzhen Hospital (Beijing, China) and involved 1,089 consecutive patients hospitalized for acute myocardial infarction (AMI) or angina pectoris (AP) who underwent OCT before PCI from January 2013 to February 2019. Of these, 539 patients were excluded based on OCT findings: 252 due to stent-related events irrelevant to native atherosclerotic lesions, 116 without pre-PCI OCT images, and 82 with poor image quality affecting plaque evaluation. 12 patients with massive thrombus and 36 with severe calcification were also excluded, as these lesions severely impaired the identification of healed plaques and plaque features. In addition, 17 patients with spontaneous coronary dissection, 11 with coronary spasm, and 13 with myocardial bridge were excluded for non-atherosclerotic etiologies. Another 106 patients were further excluded due to missing laboratory data, including 44 with unavailable hs-CRP, 10 with missing triglycerides(TG), 17 with incomplete FBG, and 35 lacking glycated albumin(GA) data. Ultimately, 444 patients were included in the final analysis.(Fig. 1). Since only a small fraction of non-culprit lesions underwent OCT assessment, our analysis was primarily focused on OCT findings of culprit lesions.

Patients’ demographic and clinical information, including age, sex, body mass index(BMI), clinical presentation, history of smoking, previous conditions include hypertension, high cholesterol, diabetes, and a family history of cardiovascular disease, as well as historical medication records and lab data at admission, were gathered from the hospital’s Electronic Medical Record (EMR) for the present study. Percutaneous coronary intervention procedure data and OCT images were obtained from the catheter Imaging System.

## Definitions

CAD includes angina pectoris, and AMI. The identification of AMI was made based on the Fourth Universal Definition of Myocardial Infarction [28], and angina was diagnosed according to the guidelines from 2020 ESC for the management of acute coronary syndromes (ACS) [29].

According to our definition, Patients with hypertension either had a history of high blood pressure, were on oral antihypertensive medication, or had a sitting blood pressure reading exceeding 140/90 mmHg at least three different occasions [30]. An individual with diabetes mellitus has a prior medical history of diabetes or a fasting blood glucose level (FBG) above 126 mg/dL, or an HbA1c level of ≥ 6.5% [31]. A patient with hyperlipidemia has low-density lipoprotein cholesterol (LDL-C) of at least 160 mg/dL or is using medication to reduce cholesterol. There were three smoking status options: never, past (≥ 100 lifetime cigarettes), or current (smoking regularly over the past 6 months). Hyperuricemia is defined as a metabolic disorder in which serum uric acid levels exceed 360 µmol/L in women and 420 µmol/L in men [32]. The diagnosis of previous myocardial infarction(MI) was made on the basis of prior documented MI, abnormal Q waves on electrocardiograms or regional wall motion irregularities. BMI=weight (kg)/height² (m²). The CTI was calculated with this formula: CTI = 0.412×ln (hs-CRP [mg/L]) + ln (TG [mg/dL] × FBG [mg/dL])/2.

### Coronary angiogram and OCT images analysis

**Fig. 2:**
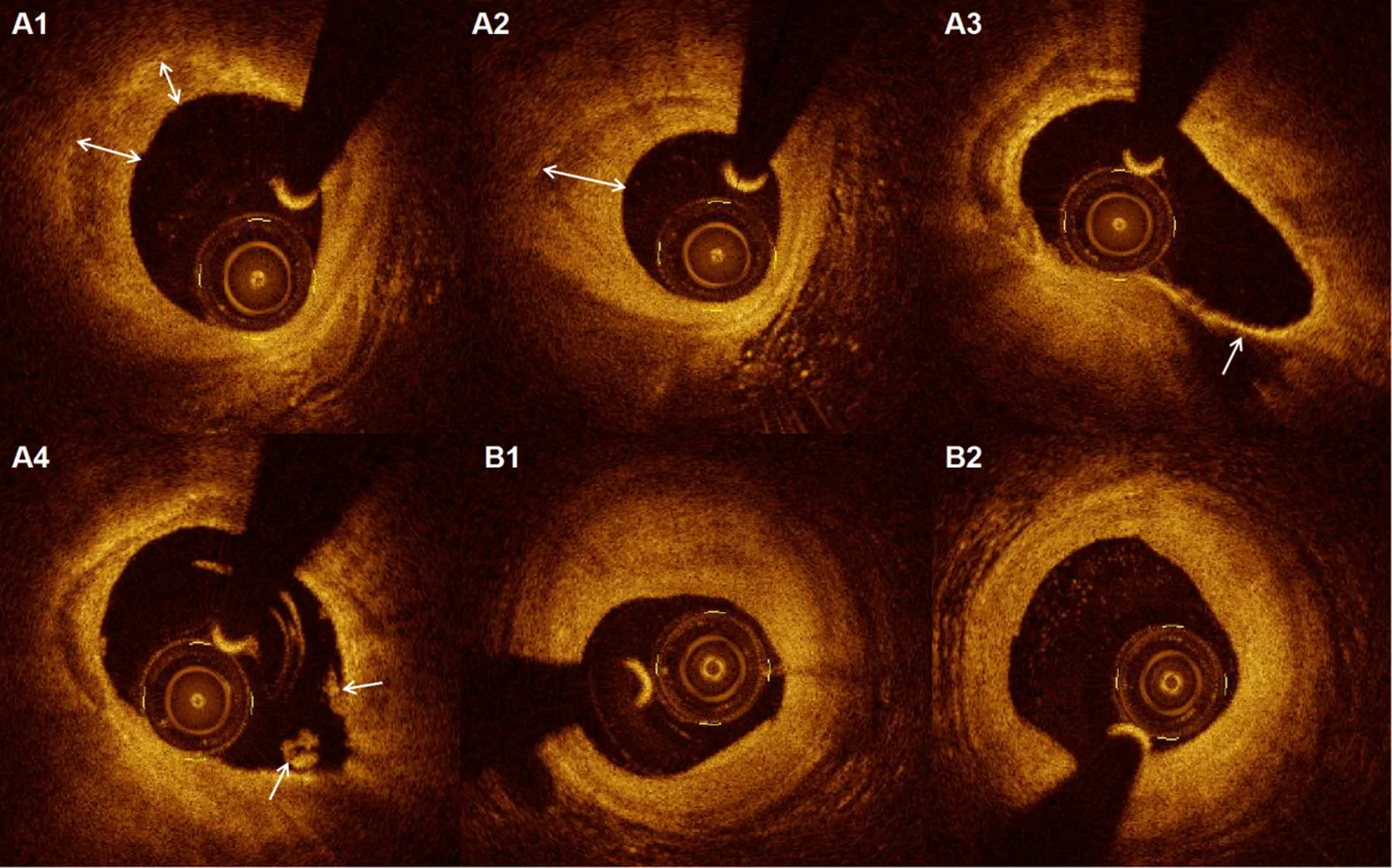
displays representative optical coherence tomography (OCT) images of coronary plaques with maximal and minimal CTI values. Panel A presents an OCT image from a patient with a high CTI value, whereas Panel B shows that from a patient with a low CTI value. Specifically, **A1** depicts multiple healed plaques, **A2** shows a single healed plaque, **A3** illustrates a thin-cap fibroatheroma (TCFA) with a thin fibrous cap, and **A4** reveals an intracoronary thrombus. In contrast, **B1** and **B2** both exhibit stable fibrous plaques

Philips Xcelera software was used to perform quantitative coronary angiography (QCA) for each angiogram. Percent diameter stenosis(minimum luminal diameter/reference vessel size x100%) and lesion length were measured. Based on the American Heart Association/American College of Cardiology classification, coronary lesions were categorized as type A, B1, B2, and C. Lesions of type B2 and C were considered complicated. Multivessel disease refers to the presence of significant diameter stenosis (≥ 50%) in two or more major coronary arteries [33]. Using certified offline software (Ilumien Optis, St. Jude Medical), professional analysts analyzed the OCT images frame by frame along the entire length of the plaque. Image analysts did not have access to the clinical data. When their evaluations were inconsistent, the imaging features were determined by a senior reviewer. The culprit plaque responsible for the patient’s clinical syndrome was identified through coronary angiography and further confirmed using the patient’s electrocardiogram, echocardiography, myocardial nuclear metabolism imaging, or cardiac magnetic resonance imaging. Definitions of OCT images were based on previous consensus [34]. Healed plaque(HP) presents with one or more layers with distinct optical densities, clearly distinguished from the components beneath. It is referred to as ‘layered plaque’ in other relevant articles. Therefore, throughout this paper, the phrases ‘healed plaque’ and ‘layered plaque’ are considered synonymously. Calcified plaques appeared as low-backscattering heterogeneous areas with clear boundaries. Cholesterol crystals were defined as line shapes with highly backscattering regions within the plaques. Macrophage accumulation was shown as signal-dense, separate or merged punctate regions with remarkable posterior shadows beneath the fibrous cap. Microvessels were presented as small black holes in the plaque, observed in at least three consecutive frames. A fibrous plaque has uniform and highly reflective regions. Lipid-rich plaques appear as areas with low signal intensity and indistinct edges. Thin-cap fibroatheroma (TCFA) is identified by a plaque rich in lipids, with the fibrous cap’s thinnest section measuring 65 μm or less and a lipid arc of at least 90°. A thrombus is characterized as an irregular, floating, or protruding mass inside the lumen, measuring at least 250 μm. (Fig. 2) Cavity formation with rupture of the fibrous cap indicates plaque rupture. We also measured the fibrous cap thickness(FCT), lipid length, and area stenosis(AS,%). The AS percentage (%) is calculated as [(average reference lumen area - smallest lumen area)/average reference lumen area] × 100. Indicators of plaque vulnerability included TCFA, macrophage buildup, plaque rupture, cholesterol crystals, and healed plaque. The kappa test was used to measure both intra- and inter-observer reproducibility. Intra-observer variability was evaluated by analyzing 80 randomly chosen plaques twice, with a two-week gap between analyses, while inter-observer variability was evaluated by two separate cardiologists at distinct timepoints. Healed plaque had intra- and inter-observer kappa values of 0.89/0.86, lipid-rich plaque had values of 0.96/0.93, calcified plaque had values of 0.95/0.94, and macrophage infiltration had values of 0.87/0.82.

### Statistical analysis

Normality tests were performed for all continuous variables prior to statistical analysis. Continuous variables, when normally distributed, were shown as mean ± standard deviation (SD) and assessed using a One-Way ANOVA test. Variables were expressed as median (25th to 75th percentiles) when the data showed a skewed distribution and analyzed applying the Kruskal-Wallis test. Categorical data were presented as frequency (percentage) and analyzed using the chi-squared test. Descriptive analysis was applied to all study participants. Restricted cubic spline (RCS) analysis was conducted to explore the relationship between CTI and lipid plaque-related parameters, including fibrous cap thickness (FCT), lipid plaque index, minimum lumen area (MLA), and lumen area stenosis (AS). For restricted cubic spline (RCS) analysis, we set 4 knots at the 5th, 35th, 65th, and 95th percentiles of the CTI distribution.

A univariate logistic regression analysis was first performed to evaluate the associations between clinical variables and coronary plaque phenotypes. Variables with a P-value < 0.1 in univariate analysis, together with established classical cardiovascular risk factors, were initially selected as potential confounders. Subsequently, we excluded individual components of the CTI index (hs-CRP, triglycerides, and fasting glucose) from further multivariable modeling to avoid multicollinearity and over-adjustment, which could otherwise distort the independent effect of the composite CTI index itself. After this exclusion, the final covariates incorporated into our logistic regression models included age, sex, BMI, smoking, hypertension, diabetes mellitus, hyperlipidemia, clinical presentation, low-density lipoprotein cholesterol (LDL-C), white blood cell (WBC), and uric acid (UA). Logistic regression models with different covariate adjustments for confounders were used to evaluate the association between CTI and the incidence of healed and vulnerable plaques. Three models were applied: Model 1 was unadjusted, Model 2 accounted for age and sex, while Model 3 included adjustments for all final selected variables. To ensure consistent results, we analyzed the association using stratified analyses. The stratification factors included diagnosis, sex, age, BMI, hypertension, diabetes, hyperlipidemia, statin use, and aspirin use.

An analysis of the receiver operating characteristic (ROC) curve was conducted to assess the discriminative values of the CTI index, WBC, LDL-C, hs-CRP, TG, and blood glucose for macrophage accumulation, cholesterol crystals, TCFA, and healed plaque. AUC, or area under the curve, was used to measure their discriminatory abilities.

Statistical analyses were conducted with R version 4.4.1 (http://www.R-project.org) and Free Statistics software version 2.0. A P-value less than 0.05 was regarded as statistically significant.

## Results

### Clinical features of the study participants

Table 1 displayed the initial features of participants divided into CTI quartiles. The average age of the 444 patients at baseline was 58.8 (± 11.7) years, and 76.6% of subjects were men (*n* = 340). The included participants were categorized into four groups according to CTI level. The mean CTI indices were 7.9 ± 0.4, 8.7 ± 0.2, 9.2 ± 0.2 and 10.1 ± 0.6 in Q1, Q2, Q3, andQ4, respectively. According to CTI quartiles, the levels of BMI, TC, LDL-C, FBG, TG, UA, WBC and GA were elevated with the increase in CTI quartiles, whereas high-density lipoprotein cholesterol(HDL-C) was decreased(*P* < 0.001). Moreover, participants in the upper CTI quartiles showed a greater incidence of diabetes.

**Table 1:** Baseline characteristics of the study population

Variables	Total (*n* = 444)	Quartile of CTI				*P*-value
		Q1 (*n* = 111)	Q2(*n* = 111)	Q3(*n* = 111)	Q4 (*n* = 111)	
Clinical presentation, n(%)						0.096
SAP	145 (32.7)	32 (28.8)	45 (40.5)	39 (35.1)	29 (26.1)	
ACS	299 (67.3)	79 (71.2)	66 (59.5)	72 (64.9)	82 (73.9)	
Age, Mean ± SD	58.8 ± 11.7	59.7 ± 11.8	59.2 ± 11.7	58.7 ± 11.9	57.8 ± 11.6	0.657
Male, n(%)	340 (76.6)	85 (76.6)	92 (82.9)	80 (72.1)	83 (74.8)	0.270
BMI, Mean ± SD	24.2 ± 2.4	23.1 ± 2.0	23.9 ± 1.8	24.4 ± 2.4	25.6 ± 2.6	< 0.001
Previous MI, n(%)	59 (13.3)	15 (13.5)	14 (12.7)	16 (14.4)	14 (12.7)	0.980
Previous PCI, n(%)	68 (15.4)	21 (18.9)	15 (13.6)	16 (14.4)	16 (14.5)	0.691
Hypertension, n(%)	237 (53.5)	56 (50.5)	55 (50)	56 (50.5)	70 (63.1)	0.142
Diabetes mellitus, n(%)	124 (28.1)	19 (17.3)	26 (23.6)	31 (27.9)	48 (43.2)	< 0.001
Hyperlipidemia, n(%)	207 (46.7)	43 (38.7)	48 (43.6)	63 (56.8)	53 (47.7)	0.050
Hyperuricemia, n(%)	18 (4.1)	2 (1.8)	4 (3.6)	6 (5.4)	6 (5.5)	0.416
Peripheral atherosclerosis, n(%)	35 (7.9)	6 (5.4)	14 (12.7)	5 (4.5)	10 (9.2)	0.092
Smoking, n(%)						0.216
None	203 (46.0)	59 (53.6)	43 (39.1)	55 (49.5)	46 (41.8)	
Past	57 (12.9)	15 (13.6)	18 (16.4)	12 (10.8)	12 (10.9)	
Current	181 (41.0)	36 (32.7)	49 (44.5)	44 (39.6)	52 (47.3)	
Family history, n(%)	88 (20.0)	19 (17.3)	21 (19.1)	25 (22.5)	23 (20.9)	0.785
Medications history at admission, n(%)						
Statin at admission	271 (61.2)	73 (65.8)	61 (55.5)	69 (62.2)	68 (61.3)	0.467
Aspirin	276 (62.4)	75 (67.6)	68 (62.4)	66 (59.5)	67 (60.4)	0.600
P_2_Y_12_ inhibitor	175 (39.6)	49 (44.1)	42 (38.5)	39 (35.1)	45 (40.5)	0.577
ACEI/ARB	79 (17.9)	22 (19.8)	15 (13.8)	25 (22.7)	17 (15.3)	0.284
Beta blocker	110 (25.1)	35 (31.8)	28 (25.7)	24 (21.8)	23 (21.1)	0.239
Laboratory data						
WBC, Mean ± SD	6.6 ± 1.7	6.0 ± 1.6	6.5 ± 1.7	6.6 ± 1.5	7.3 ± 1.7	< 0.001
LDL-C, Mean ± SD	2.5 ± 1.0	2.1 ± 0.9	2.5 ± 0.9	2.6 ± 1.2	2.6 ± 0.9	< 0.001
TC, Mean ± SD	4.1 ± 1.2	3.7 ± 1.1	4.1 ± 1.0	4.3 ± 1.3	4.5 ± 1.2	< 0.001
Triglycerides, Mean ± SD	1.8 ± 1.6	1.0 ± 0.3	1.5 ± 0.5	1.9 ± 0.8	2.9 ± 2.6	< 0.001
HDL-C, Mean ± SD	1.0 ± 0.3	1.2 ± 0.3	1.0 ± 0.2	1.0 ± 0.4	0.9 ± 0.2	< 0.001
Hcy, Mean ± SD	15.5 ± 10.2	15.1 ± 9.9	15.9 ± 10.1	14.1 ± 7.9	16.8 ± 12.7	0.266
FBG, Mean ± SD	6.3 ± 2.2	5.4 ± 1.1	6.0 ± 1.6	6.1 ± 1.9	7.6 ± 3.0	< 0.001
Creatinine, Mean ± SD	72.6 ± 13.4	71.6 ± 13.5	73.7 ± 12.9	72.0 ± 13.8	73.2 ± 13.6	0.619
UA, Mean ± SD	355.3 ± 86.8	334.2 ± 69.5	345.3 ± 83.8	360.6 ± 94.5	381.1 ± 91.3	< 0.001
hsCRP, Mean ± SD	15.0 ± 3.6	14.3 ± 3.1	14.6 ± 2.9	14.7 ± 3.2	16.5 ± 4.7	< 0.001
GA,%	9.0 ± 0.9	7.9 ± 0.4	8.7 ± 0.2	9.2 ± 0.2	10.1 ± 0.6	< 0.001
CTI, Mean ± SD	9.0 ± 0.9	7.9 ± 0.4	8.7 ± 0.2	9.2 ± 0.2	10.1 ± 0.6	< 0.001

SAP, stable angina pectoris; UAP, unstable angina pectoris; NSTEMI, Non-ST-Segment Elevation Myocardial Infarction; STEMI, ST-Segment Elevation Myocardial Infarction; BMI, body mass index; MI, Myocardial Infarction; PCI, Percutaneous Coronary Intervention; ACEI, angiotensin-converting enzyme inhibitor; ARB, angiotensin II receptor blocker; LDL-C, low-density lipoprotein cholesterol; TC, total cholesterol; GA, Glycated albumin; HDL-C, high-density lipoprotein cholesterol; Hcy, Homocysteine; FBG, fast blood glucose; UA, Uric Acid; hs-CRP, high-sensitive C-reactive protein; CTI, high-sensitive C-reactive protein-triglyceride-glucose index; SD, Standard Deviation

### Coronary findings based on CTI quartile groups

The angiographic data were summarized in Table 2. There was a stepwise increase in lesion length across the ascending CTI quartiles. Regarding multi-vessel disease (MVD), its prevalence showed a progressive rise with higher CTI quartiles. The incidence of MVD was 20.7% in Q1, 21.8% in Q2, 36.0% in Q3, and 45.0% in Q4 (*P* < 0.001) (Table 2). Complex lesions, including type B2 and type C, were significantly more frequent in the Q3 and Q4 quartiles of CTI than in the Q1 and Q2 quartiles(46.8–51.8% vs. 30.3–33.3%).

**Table 2:** Angiographic characteristics of the population

Variables	Total (*n* = 444)	Quartile of CTI				*P*-value
		Q1 (*n* = 111)	Q2(*n* = 111)	Q3(*n* = 111)	Q4 (*n* = 111)	
OCT vessel, n (%)						0.864
LAD	294 (66.2)	74 (66.7)	69 (62.2)	76 (68.5)	75 (67.6)	
LCX	53 (11.9)	11 (9.9)	15 (13.5)	15 (13.5)	12 (10.8)	
RCA	97 (21.8)	26 (23.4)	27 (24.3)	20 (18)	24 (21.6)	
Lesion length, Mean ± SD	18.3 ± 8.5	16.0 ± 7.1	17.9 ± 7.8	18.1 ± 8.0	21.4 ± 10.0	< 0.001
MLD, Mean ± SD	1.5 ± 0.5	1.6 ± 0.6	1.5 ± 0.5	1.5 ± 0.5	1.4 ± 0.4	0.317
RVD, Mean ± SD	3.8 ± 0.6	3.9 ± 0.6	3.8 ± 0.6	3.9 ± 0.6	3.9 ± 0.6	0.579
DS, Mean ± SD	61.5 ± 11.4	60.0 ± 12.3	61.1 ± 10.2	62.2 ± 12.6	62.8 ± 9.9	0.347
Multivessel disease, n (%)	137 (30.9)	23 (20.7)	24 (21.8)	40 (36)	50 (45)	< 0.001
Type B2 + Type C, n (%)	179 (40.6)	37 (33.3)	33 (30.3)	52 (46.8)	57 (51.8)	0.002

LAD, left anterior descending artery; LCX, left circumflex artery; RCA, right coronary artery; MLD, minimal lumen diameter; RVD, reference vessel diameter; DS, diameter stenosis; CTI, high-sensitive C-reactive protein-triglyceride-glucose index; SD, Standard Deviation

### Morphological characteristics of OCT findings

**Fig. 3:**
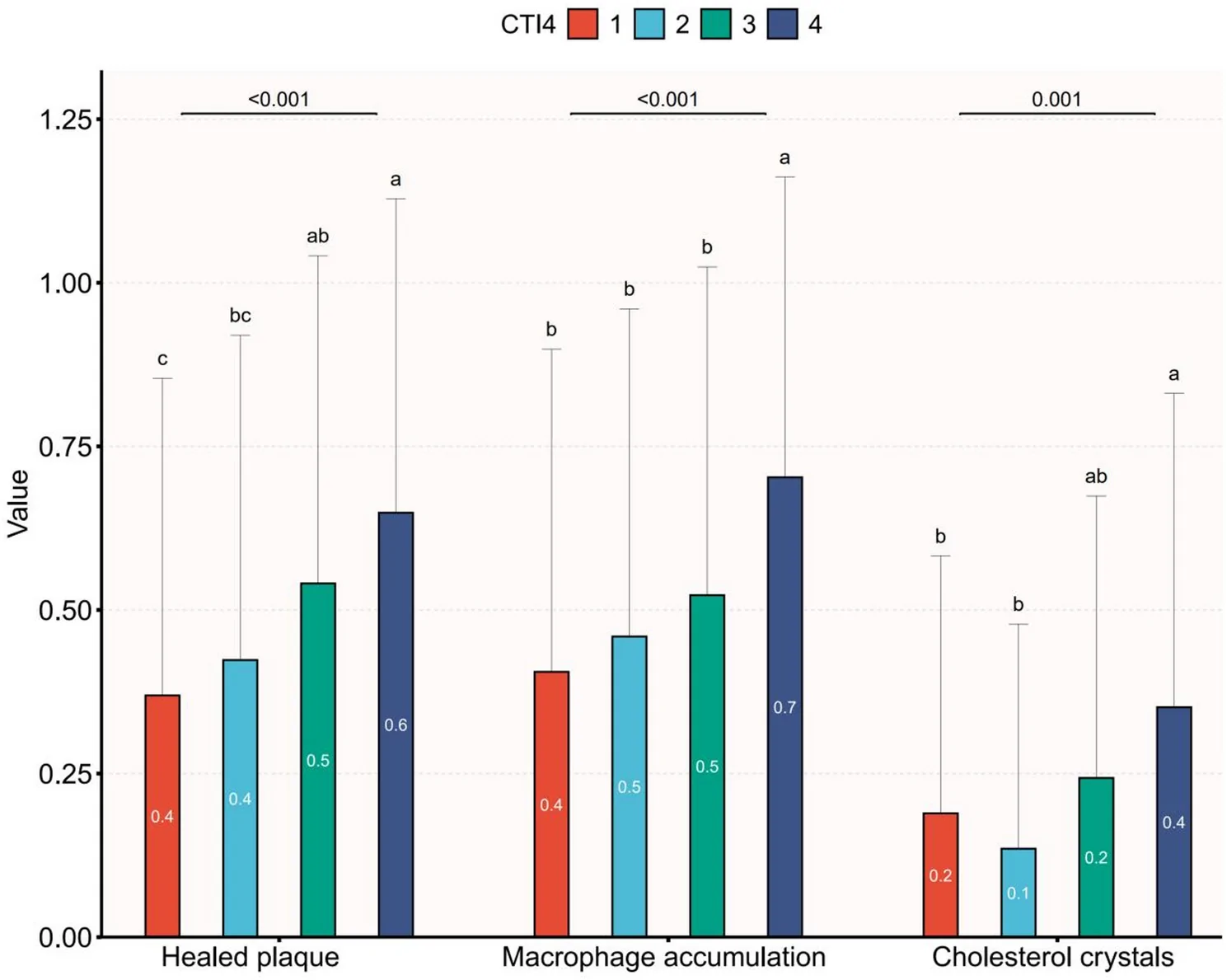
Bar graph showing the optical coherence tomography (OCT) findings of coronary plaques across four CTI quartile groups. A significant upward trend in the incidence of healed plaques, macrophage infiltration, and cholesterol crystals was observed with increasing CTI quartiles

Table 3 showed the morphological results of OCT observations grouped according to CTI quartiles. Patients with a high CTI exhibited more characteristics of high-risk and vulnerable coronary plaques. As the CTI quartiles increased, the incidence rates of macrophage accumulation and healed plaque gradually rose (macrophage aggregation: 40.5%, 45.9%, 58.0%, 78.0%; healed plaque: 36.9%, 42.3%, 54.1%, 64.9%, respectively, from Q1 to Q4). In addition, compared with the low CTI groups (Q1 and Q2), the incidences of cholesterol crystals and multiple healed plaques (≥ 2) were significantly higher in the high CTI groups (Q3 and Q4). The percentages for cholesterol crystals were 24.3% and 35.1% in Q3 and Q4, respectively, compared to 18.9% and 13.5% in Q1 and Q2. For multiple healed plaques, the incidences were 18.0% and 18.9% in Q3 and Q4, respectively, versus 12.6% in both Q1 and Q2. (Fig. 3). A similar trend was observed in terms of plaque rupture, whereas no significant differences were noted in other aspects, such as calcified plaques.

**Table 3:** Morphological characteristics by optical coherence tomography

Variables	Total (*n* = 444)	Quartile of CTI				*P*-value
		Q1 (*n* = 111)	Q2(*n* = 111)	Q3(*n* = 111)	Q4 (*n* = 111)	
Fibrous plaque, n (%)	299 (67.3)	75 (67.6)	74 (66.7)	80 (72.1)	70 (63.1)	0.556
Lipid-rich plaque, n (%)	383 (86.3)	88 (79.3)	96 (86.5)	97 (87.4)	102 (91.9)	0.054
TCFA, n (%)	82 (18.5)	17 (15.3)	13 (11.7)	25 (22.5)	27 (24.3)	0.049
FCT, Mean ± SD	93.6 ± 49.7	107.1 ± 77.2	98.3 ± 44.4	86.1 ± 3 3.5	84.2 ± 30.5	0.003
Lipid length, Mean ± SD	7.8 ± 3.1	7.4 ± 3.0	7.2 ± 2.5	7.5 ± 3.2	8.8 ± 3.3	< 0.001
Mean lipid arc, Mean ± SD	208.7 ± 66.3	195.9 ± 56.9	196.0 ± 61.5	220.1 ± 73.1	221.0 ± 68.1	0.004
Lipid index, Mean ± SD	1671.5 ± 941.8	1487.3 ± 833.2	1454.5 ± 760.7	1719.6 ± 1043.1	1992.2 ± 999.9	< 0.001
Max lipid arc, Mean ± SD	242.3 ± 73.3	233.4 ± 68.5	230.2 ± 72.4	249.6 ± 77.2	257.7 ± 72.7	0.054
Calcification, n (%)	235 (52.9)	54 (48.6)	54 (48.6)	61 (55)	66 (59.5)	0.294
Cholesterol crystals, n (%)	102 (23.0)	21 (18.9)	15 (13.5)	27 (24.3)	39 (35.1)	0.001
Microvessels, n (%)	162 (36.7)	42 (37.8)	38 (34.2)	43 (38.7)	39 (35.8)	0.899
Macrophage accumulation, n (%)	232 (52.3)	45 (40.5)	51 (45.9)	58 (52.3)	78 (70.3)	< 0.001
Healed plaque, n (%)	220 (49.5)	41 (36.9)	47 (42.3)	60 (54.1)	72 (64.9)	< 0.001
Healed plaque number, n (%)						0.002
0	225 (50.7)	70 (63.1)	64 (57.7)	52 (46.8)	39 (35.1)	
1	150 (33.8)	27 (24.3)	33 (29.7)	39 (35.1)	51 (45.9)	
≥ 2	69 (15.5)	14 (12.6)	14 (12.6)	20 (18)	21 (18.9)	
Plaque rupture, n (%)	81 (18.2)	14 (12.6)	15 (13.5)	26 (23.4)	26 (23.4)	0.046
MLA, Mean ± SD	2.6 ± 1.7	3.0 ± 2.0	2.5 ± 1.5	2.5 ± 1.6	2.3 ± 1.4	0.014
RLA, Mean ± SD	9.7 ± 3.1	9.5 ± 3.1	9.5 ± 2.9	10.0 ± 3.3	9.9 ± 3.0	0.518
AS, Mean ± SD	73.7 ± 12.8	70.0 ± 14.3	73.7 ± 11.8	74.5 ± 12.5	76.5 ± 11.7	0.002

TCFA, thin cap fibroatheroma; FCT, fibrous cap thickness; MLA, minimal lumen area; RLA, reference lumen area; AS, area stenosis; CTI, high-sensitive C-reactive protein-triglyceride-glucose index; SD, Standard Deviation

To further clarify whether such associations differed by clinical presentation, we stratified the overall cohort into stable angina pectoris (SAP, *n* = 145) and acute coronary syndrome (ACS, *n* = 299) subgroups and re-analyzed OCT morphological characteristics across CTI quartiles (Tables 4 and 5).

**Table 4:** Morphological characteristics by optical coherence tomography in SAP patients

Variables	Total	Quartile of CTI				*P*-value
	(*n* = 145)	Q1 (*n* = 36)	Q2(*n* = 36)	Q3(*n* = 36)	Q4 (*n* = 37)	
Fibrous plaque, n (%)	97 (66.9)	26 (72.2)	23 (63.9)	21 (58.3)	27 (73)	0.49
Lipid-rich plaque, n (%)	130 (89.7)	31 (86.1)	32 (88.9)	33 (91.7)	34 (91.9)	0.844
TCFA, n (%)	21 (14.5)	5 (13.9)	3 (8.3)	6 (16.7)	7 (18.9)	0.607
FCT, Mean ± SD	92.7 ± 33.1	86.8 ± 29.0	103.9 ± 41.4	89.9 ± 31.6	89.7 ± 27.4	0.154
Lipid length, Mean ± SD	8.1 ± 3.3	8.6 ± 3.3	7.5 ± 2.7	7.4 ± 3.1	9.1 ± 3.8	0.117
Mean lipid arc, Mean ± SD	190.0 ± 49.2	197.6 ± 53.8	177.5 ± 50.0	189.5 ± 47.8	195.9 ± 45.3	0.345
Lipid index, Mean ± SD	1589.4 ± 860.1	1735.9 ± 1002.9	1374.1 ± 694.3	1442.2 ± 809.5	1811.8 ± 875.4	0.102
Max lipid arc, Mean ± SD	239.8 ± 70.2	246.6 ± 71.3	219.9 ± 71.5	245.2 ± 73.4	247.7 ± 64.0	0.315
Calcification, n (%)	90 (62.1)	22 (61.1)	19 (52.8)	25 (69.4)	24 (64.9)	0.515
Cholesterol crystals, n (%)	46 (31.7)	14 (38.9)	7 (19.4)	10 (27.8)	15 (40.5)	0.176
Microvessels, n (%)	51 (35.2)	15 (41.7)	13 (36.1)	12 (33.3)	11 (29.7)	0.75
Macrophage accumulation, n (%)	74 (51.0)	16 (44.4)	19 (52.8)	17 (47.2)	22 (59.5)	0.587
Healed plaque, n (%)	90 (62.1)	24 (66.7)	18 (50)	21 (58.3)	27 (73)	0.201
Healed plaque number, n (%)						0.198
0	55 (37.9)	12 (33.3)	18 (50)	15 (41.7)	10 (27)	
1	53 (36.6)	15 (41.7)	14 (38.9)	10 (27.8)	14 (37.8)	
≥ 2	37 (25.5)	9 (25)	4 (11.1)	11 (30.6)	13 (35.1)	
Plaque rupture, n (%)	14 (9.7)	6 (16.7)	5 (13.9)	2 (5.6)	1 (2.7)	0.137
MLA, Mean ± SD	2.4 ± 1.5	2.2 ± 1.6	2.4 ± 1.2	2.7 ± 1.8	2.3 ± 1.3	0.571
RLA, Mean ± SD	9.4 ± 3.1	8.5 ± 2.6	9.0 ± 2.5	10.3 ± 3.9	9.8 ± 2.9	0.057
AS, Mean ± SD	74.9 ± 11.4	74.6 ± 14.1	73.5 ± 10.6	74.7 ± 10.2	76.9 ± 10.4	0.631

TCFA, thin cap fibroatheroma; FCT, fibrous cap thickness; MLA, minimal lumen area; RLA, reference lumen area; AS, area stenosis; CTI, high-sensitive C-reactive protein-triglyceride-glucose index; SD, Standard Deviation

**Table 5:** Morphological characteristics by optical coherence tomography in ACS patients

Variables	Total	Quartile of CTI				*P*-value
	(*n* = 299)	Q1 (*n* = 75)	Q2(*n* = 74)	Q3(*n* = 75)	Q4 (*n* = 75)	
Fibrous plaque, n (%)	202 (67.6)	50 (66.7)	50 (67.6)	54 (72)	48 (64)	0.768
Lipid-rich plaque, n (%)	253 (84.6)	56 (74.7)	64 (86.5)	65 (86.7)	68 (90.7)	0.041
TCFA, n (%)	61 (20.4)	12 (16)	10 (13.5)	20 (26.7)	19 (25.3)	0.112
FCT, Mean ± SD	94.0 ± 56.1	114.8 ± 90.5	96.2 ± 47.9	83.8 ± 35.1	83.9 ± 32.5	0.005
Lipid length, Mean ± SD	7.6 ± 2.9	6.8 ± 2.8	7.1 ± 2.4	7.6 ± 3.1	8.6 ± 3.2	0.003
Mean lipid arc, Mean ± SD	218.2 ± 71.8	199.4 ± 60.2	204.6 ± 67.4	235.6 ± 75.5	230.1 ± 76.1	0.007
Lipid index, Mean ± SD	1713.2 ± 979.6	1377.9 ± 720.7	1503.4 ± 794.6	1874.3 ± 1087.3	2040.2 ± 1093.8	< 0.001
Max lipid arc, Mean ± SD	244.1 ± 75.5	227.3 ± 67.3	235.1 ± 72.7	262.0 ± 81.0	254.4 ± 78.2	0.092
Calcification, n (%)	145 (48.5)	34 (45.3)	31 (41.9)	38 (50.7)	42 (56)	0.331
Cholesterol crystals, n (%)	56 (18.7)	8 (10.7)	7 (9.5)	16 (21.3)	25 (33.3)	< 0.001
Microvessels, n (%)	111 (37.4)	30 (40)	20 (27)	32 (42.7)	29 (39.7)	0.197
Macrophage accumulation, n (%)	158 (52.8)	29 (38.7)	34 (45.9)	43 (57.3)	52 (69.3)	0.001
Healed plaque, n (%)	130 (43.5)	17 (22.7)	28 (37.8)	41 (54.7)	44 (58.7)	< 0.001
Healed plaque number, n (%)						< 0.001
0	170 (56.9)	58 (77.3)	46 (62.2)	35 (46.7)	31 (41.3)	
1	97 (32.4)	13 (17.3)	18 (24.3)	31 (41.3)	35 (46.7)	
≥ 2	32 (10.7)	4 (5.3)	10 (13.5)	9 (12)	9 (12)	
Plaque rupture, n (%)	67 (22.4)	9 (12)	10 (13.5)	25 (33.3)	23 (30.7)	0.001
MLA, Mean ± SD	2.7 ± 1.7	3.2 ± 2.0	2.7 ± 1.7	2.5 ± 1.7	2.3 ± 1.5	0.006
RLA, Mean ± SD	9.9 ± 3.0	10.0 ± 3.1	10.0 ± 3.1	9.8 ± 3.0	10.0 ± 3.0	0.987
AS, Mean ± SD	73.0 ± 13.4	68.1 ± 13.9	73.5 ± 12.8	74.5 ± 13.5	76.0 ± 12.4	0.002

TCFA, thin cap fibroatheroma; FCT, fibrous cap thickness; MLA, minimal lumen area; RLA, reference lumen area; AS, area stenosis; CTI, high-sensitive C-reactive protein-triglyceride-glucose index; SD, Standard Deviation

In the ACS subgroup, the graded positive associations between elevated CTI and vulnerable plaque phenotypes were highly consistent with the overall population findings. By contrast, in the SAP subgroup, none of the vulnerable plaque features demonstrated a significant graded association with CTI quartiles (all P for trend > 0.05).

### Correlation of CTI and lipid plaque-related parameters and lumen area

**Fig. 4:**
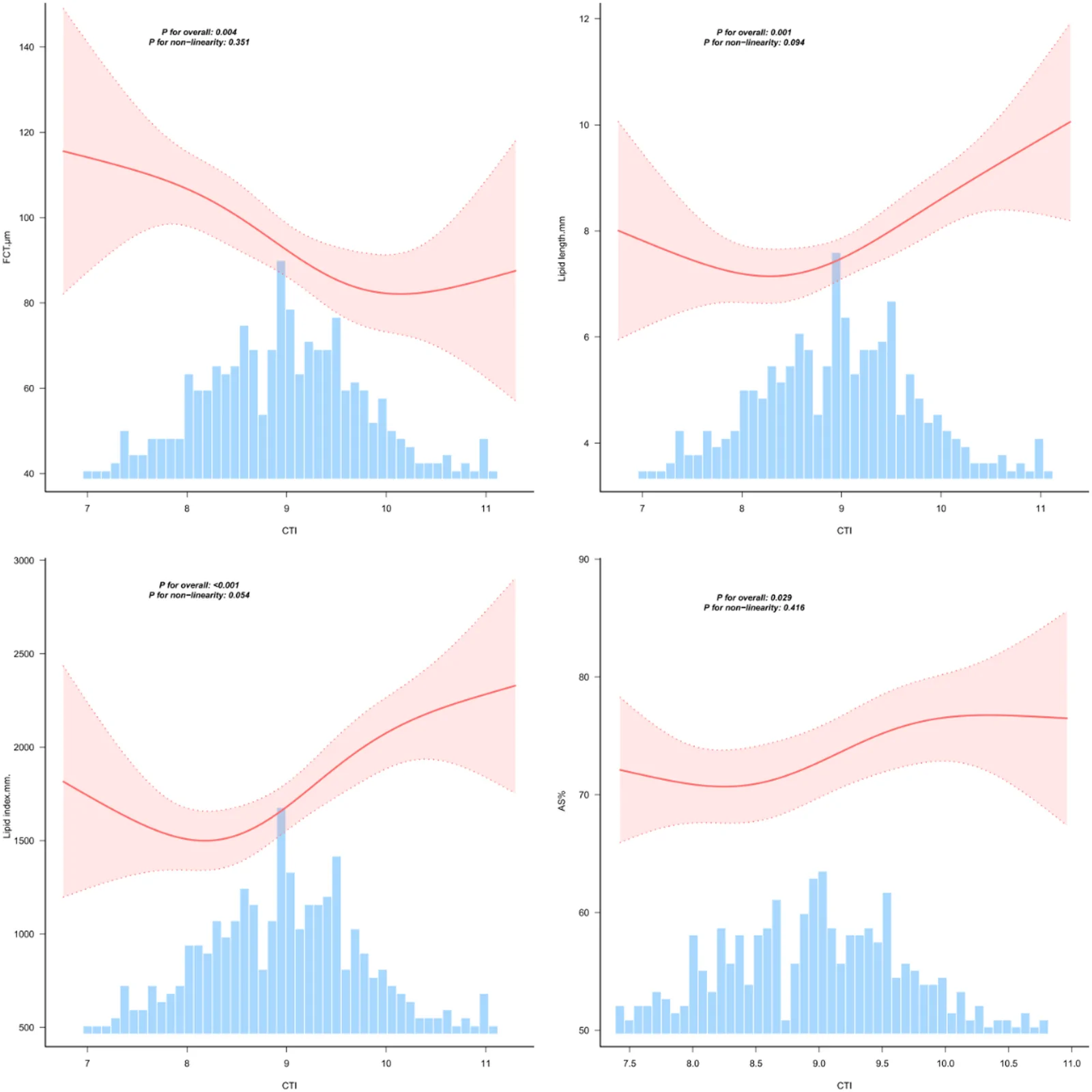
RCS analysis of the association between CTI and various lipid plaque-related parameters(fibrous cap thickness (FCT), lipid length, lipid plaque index, area stenosis)

The RCS analysis showed a linear association between CTI and various lipid plaque-related parameters (all *P* < 0.05), with no evidence of nonlinearity(Fig. 4). As the CTI quartile increased, the fibrous cap thickness (FCT) of lipid plaques gradually decreased (P for non-linearity = 0.351), while the lipid length and lipid plaque index showed a progressive elevation.

Additionally, the minimal lumen area was negatively associated with increasing CTI quantiles (CTI from Q1 to Q4; MLA values of 3.0, 2.5, 2.5, and 2.3 mm², respectively; *P* = 0.014). Consistent with this, the rate of area stenosis (AS) exhibited a progressive elevation with ascending CTI quartiles, increasing from 70.0% in Q1 to 76.8% in Q4 (*P* = 0.002). (Table 3)

### Correlation between CTI and coronary plaques

The correlation between CTI parameters and coronary plaque characteristics was investigated using multiple logistic regression models(Table 6). After covariate adjustment in model 3, each unit increase in CTI was significantly correlated with a greater probability of healed plaque (OR = 1.62, 95% CI: 1.21–2.16, *P* = 0.001), cholesterol crystals (OR = 1.64, 95% CI: 1.17–2.30, *P* = 0.004), macrophage accumulation (OR = 1.49, 95% CI: 1.13–1.98, *P* = 0.005), and plaque rupture (OR = 1.54, 95% CI: 1.07–2.21, *P* = 0.019).

When CTI parameters were categorized into quartiles (Q1 as the reference group), the adjusted associations (model 3) showed that the Q4 group remained significantly correlated with the following plaque characteristics compared to the Q1 group: healed plaque (OR = 2.54, 95% CI [1.32–4.49], *P* = 0.006), macrophage infiltration (OR = 2.78, 95% CI [1.45–5.33], *P* = 0.002), and plaque rupture (OR = 2.84, 95% CI [1.16–6.93], *P* = 0.022).

**Table 6:** Association of CTI with coronary plaque in multiple regression model

	Model 1		Model 2		Model 3	
	OR (95% CI)	*P*-value	OR (95% CI)	*P*-value	OR (95% CI)	*P*-value
Healed plaque						
CTI (per unit)	1.71 (1.36 ~ 2.16)	< 0.001	1.73 (1.37 ~ 2.19)	< 0.001	1.62 (1.21 ~ 2.16)	0.001
Q1	1(Ref)		1(Ref)		1(Ref)	
Q2	1.25 (0.73 ~ 2.15)	0.411	1.23 (0.72 ~ 2.12)	0.447	1 (0.56 ~ 1.79)	0.995
Q3	2.01 (1.17 ~ 3.44)	0.011	2.05 (1.2 ~ 3.51)	0.009	1.64 (0.9 ~ 2.98)	0.104
Q4	3.15 (1.82 ~ 5.45)	< 0.001	3.21 (1.85 ~ 5.56)	< 0.001	2.54 (1.32 ~ 4.91)	0.006
**Cholesterol crystals**						
CTI (per unit)	1.6 (1.24 ~ 2.07)	< 0.001	1.66 (1.28 ~ 2.16)	< 0.001	1.64 (1.17 ~ 2.3)	0.004
Q1	1(Ref)		1(Ref)		1(Ref)	
Q2	0.67 (0.33 ~ 1.38)	0.277	0.66 (0.32 ~ 1.37)	0.264	0.49 (0.23 ~ 1.07)	0.072
Q3	1.38 (0.72 ~ 2.62)	0.329	1.44 (0.75 ~ 2.75)	0.274	1.24 (0.61 ~ 2.51)	0.551
Q4	2.32 (1.26 ~ 4.29)	0.007	2.46 (1.32 ~ 4.57)	0.005	2.09 (0.99 ~ 4.43)	0.053
**Macrophage accumulation**						
CTI (per unit)	1.67 (1.32 ~ 2.1)	< 0.001	1.65 (1.31 ~ 2.08)	< 0.001	1.49 (1.13 ~ 1.98)	0.005
Q1	1(Ref)		1(Ref)		1(Ref)	
Q2	1.25 (0.73 ~ 2.12)	0.417	1.24 (0.73 ~ 2.11)	0.431	1.13 (0.64 ~ 1.98)	0.668
Q3	1.61 (0.94 ~ 2.73)	0.081	1.59 (0.93 ~ 2.71)	0.087	1.34 (0.75 ~ 2.39)	0.319
Q4	3.47 (1.99 ~ 6.05)	< 0.001	3.41 (1.95 ~ 5.96)	< 0.001	2.78 (1.45 ~ 5.33)	0.002
**Plaque rupture**						
CTI (per unit)	1.42 (1.08 ~ 1.87)	0.012	1.39 (1.06 ~ 1.83)	0.018	1.54 (1.07 ~ 2.21)	0.019
Q1	1(Ref)		1(Ref)		1(Ref)	
Q2	1.08 (0.5 ~ 2.36)	0.842	1.05 (0.48 ~ 2.3)	0.908	1.27 (0.54 ~ 2.99)	0.582
Q3	2.12 (1.04 ~ 4.32)	0.039	2.11 (1.03 ~ 4.33)	0.041	2.97 (1.31 ~ 6.74)	0.009
Q4	2.12 (1.04 ~ 4.32)	0.039	2.05 (1 ~ 4.2)	0.05	2.84 (1.16 ~ 6.93)	0.022

Model 1: unadjusted

Model 2: adjusted for age and sex

Model 3: adjusted for age, sex, BMI, smoking, clinical presentation, hypertension, diabetes mellitus, hyperlipidemia, hyperuricemia, low-density lipoprotein cholesterol, WBC and UA levels

CTI, high-sensitive C-reactive protein-triglyceride-glucose index; WBC, white blood cell; UA, Uric Acid; OR, odds ratio; CI, confidence interval; Ref, reference

### Stratified logistic regression analyses by clinical presentation

Stratified logistic regression analyses were performed to evaluate the associations of CTI with coronary plaque phenotypes stratified by clinical presentation, accompanied by formal interaction testing (Table 7).

**Table 7:** Subgroup Analysis and Interaction Testing of CTI and Coronary Plaque Phenotypes by Clinical Presentation

Phenotype/Subgroup	Total *n*	Adjusted OR (95% CI)	*P*-value	*P* for Interaction
Healed plaque				0.067
SAP	145	1.36 (0.78 ~ 2.38)	0.285	
ACS	299	1.7 (1.19 ~ 2.44)	0.004	
Cholesterol crystals				0.037
SAP	145	1.18 (0.66 ~ 2.09)	0.581	
ACS	299	1.7 (1.09 ~ 2.66)	0.02	
Macrophage accumulation				0.322
SAP	145	1.17 (0.68 ~ 2.03)	0.575	
ACS	299	1.6 (1.14 ~ 2.24)	0.007	
Plaque rupture				0.006
SAP	145	0.76 (0.28 ~ 2.11)	0.603	
ACS	299	1.75 (1.17 ~ 2.61)	0.006	

CTI, high-sensitive C-reactive protein-triglyceride-glucose index; SAP, Stable Angina Pectoris; ACS, Acute Coronary Syndrome; OR, Odds Ratio; CI, Confidence Interval

After comprehensive covariate adjustment, per-unit increments in CTI were independently associated with increased odds of healed plaque, cholesterol crystals, macrophage accumulation, and plaque rupture only in patients with ACS. In contrast, no significant independent associations between CTI and any coronary plaque phenotype were observed among patients with SAP (all *P* > 0.05).

Formal interaction testing further confirmed significant effect modification by clinical presentation for cholesterol crystals (P for interaction = 0.037) and plaque rupture (P for interaction = 0.006). A borderline significant interaction was identified for healed plaques (P for interaction = 0.067), whereas no meaningful interaction was detected for macrophage accumulation (P for interaction = 0.322).

### Analysis of subgroups regarding the connection between CTI with coronary plaques

**Fig. 5:**
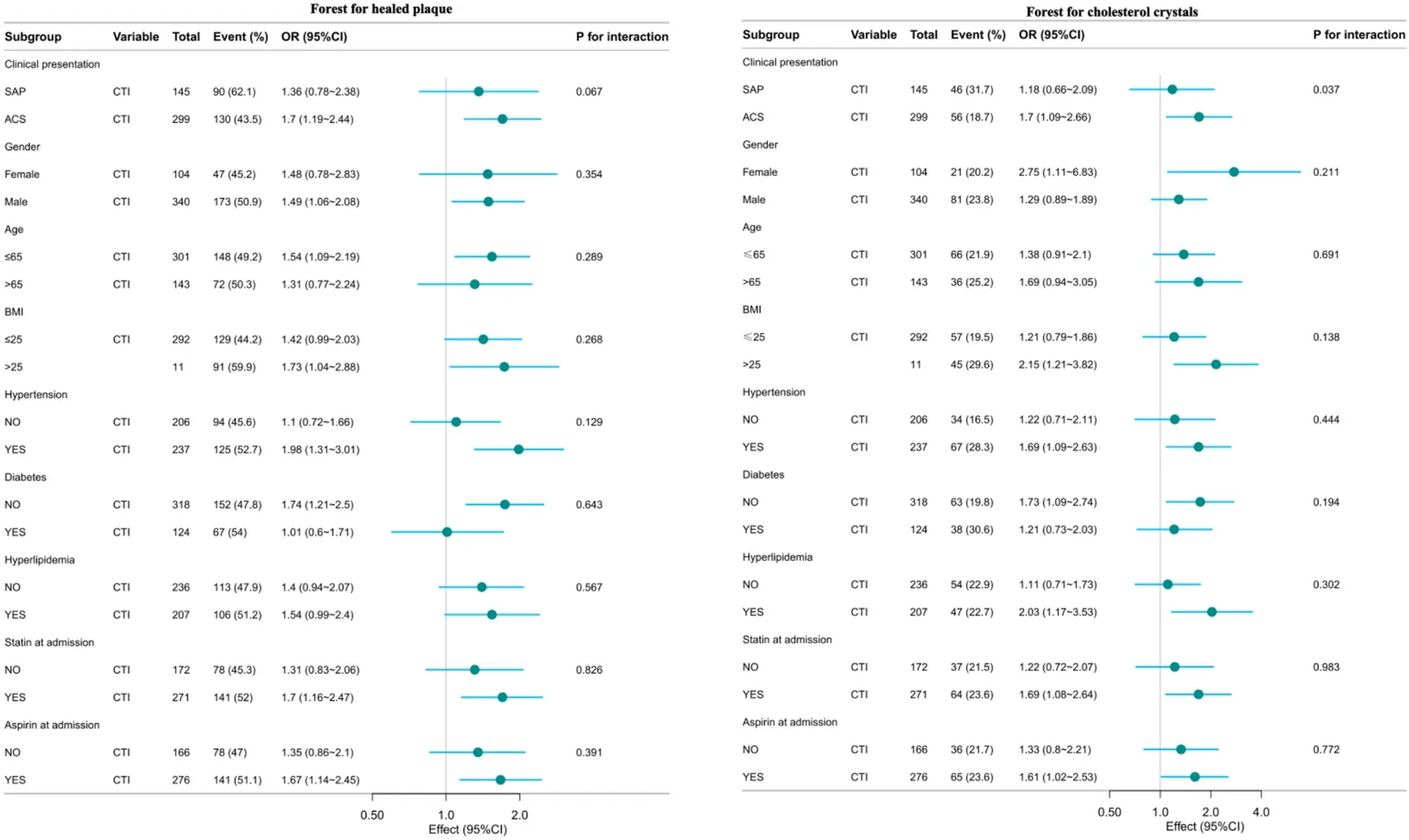

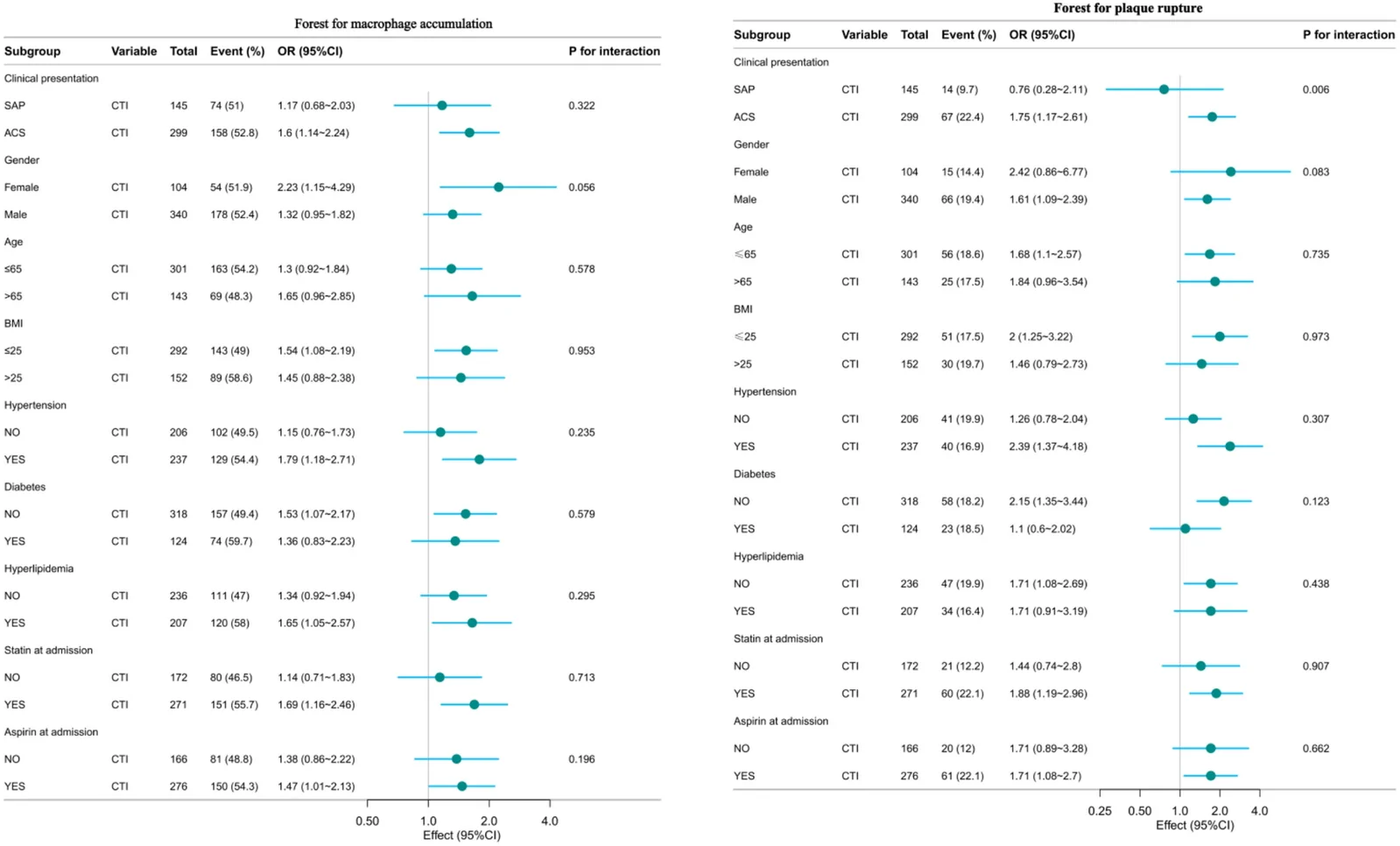
Forest plot of the CTI association with healed plaque, cholesterol crystals, macrophage accumulation and plaque rupture using logistic regression model

Subgroup analyses (Fig. 5) demonstrated that the associations between CTI and plaque phenotypes differed by clinical presentation (SAP vs. ACS), consistent with our stratified logistic regression findings above. No differences were observed across subgroups stratified by sex, age, BMI, hypertension, diabetes, hyperlipidemia, statin use, or aspirin therapy (all P for interaction > 0.05).

### Discriminative performance of CTI for coronary plaque vulnerability features

**Fig. 6:**
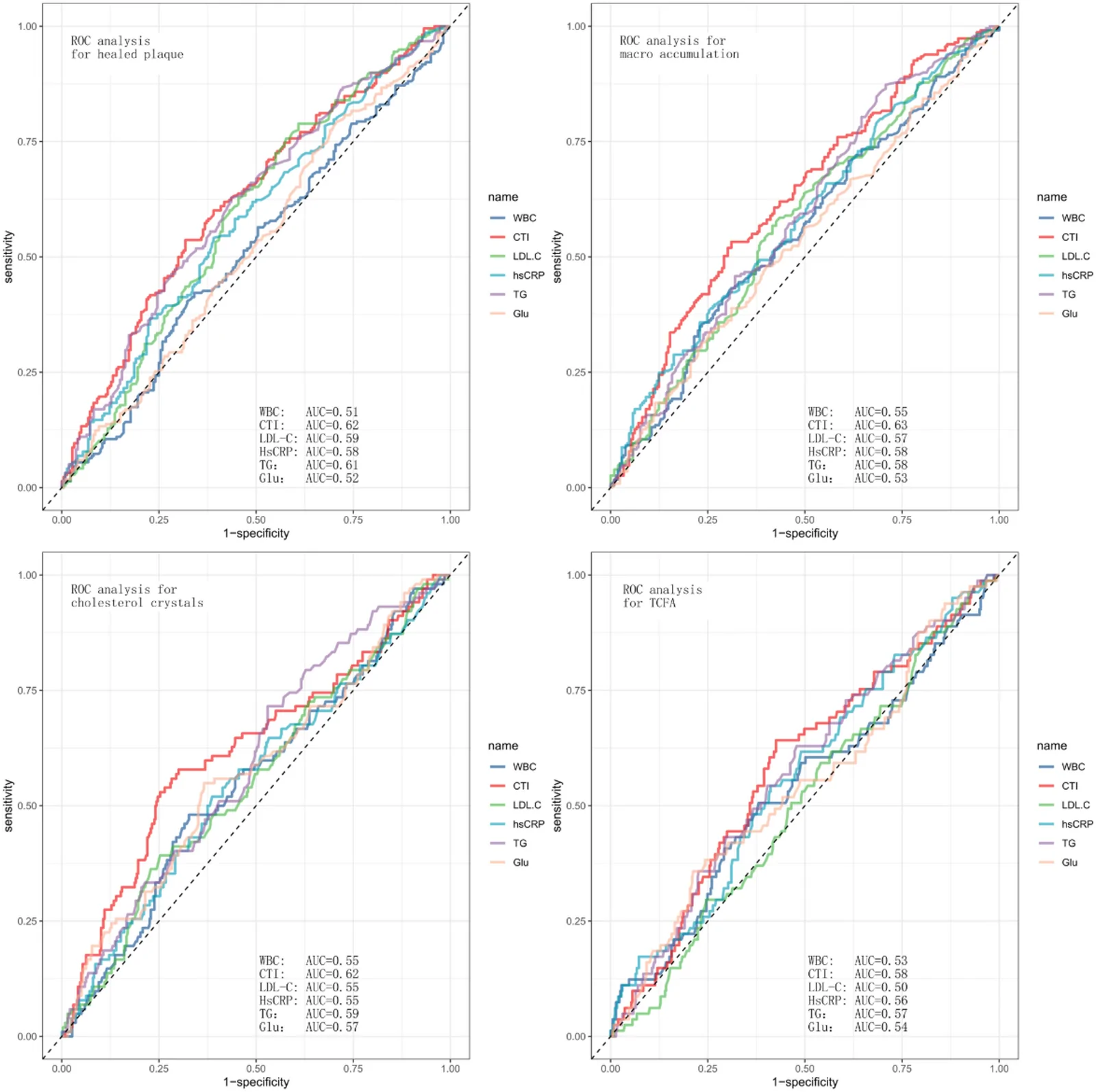
ROC curve analysis for prediction of vulnerable plaques. Figure illustrate the comparative discriminative performance of CTI and other factors for macrophage accumulation, cholesterol crystals, thin-cap fibroatheroma (TCFA), and healed plaques, respectively

ROC curve analyses (Table 8; Fig. 6) demonstrated that among all individual biomarkers assessed, CTI yielded the highest AUC values across the four vulnerable coronary plaque phenotypes, including healed plaque (AUC = 0.62), macrophage accumulation (AUC = 0.63), cholesterol crystals (AUC = 0.62), and thin-cap fibroatheroma (TCFA, AUC = 0.58). Pairwise comparisons further revealed that CTI exhibited significantly superior discriminative capacity for most plaque phenotypes compared with white blood cell count and fasting glucose.

**Table 8:** ROC analysis of CTI and conventional biomarkers for coronary plaque vulnerable phenotypes

Biomarker	Healed plaque			Macrophage accumulation			Cholesterol crystals			TCFA		
	AUC	95% CI	*P* value	AUC	95% CI	*P* value	AUC	95% CI	*P* value	AUC	95% CI	*P* value
CTI	0.62	0.58–0.68	Ref.	0.63	0.58–0.68	Ref.	0.62	0.56–0.69	Ref.	0.58	0.51–0.65	Ref.
WBC	0.51	0.46–0.57	< 0.001	0.55	0.49–0.61	0.03	0.55	0.49–0.62	0.07	0.53	0.46–0.61	0.28
LDL-C	0.59	0.54–0.64	0.26	0.57	0.52–0.62	0.38	0.55	0.48–0.61	0.07	0.5	0.44–0.57	0.10
hs-CRP	0.58	0.53–0.64	0.05	0.58	0.53–0.64	0.03	0.56	0.49–0.62	0.005	0.56	0.49–0.63	0.41
TG	0.61	0.56–0.67	0.52	0.58	0.53–0.63	0.06	0.59	0.53–0.65	0.26	0.57	0.51–0.65	0.93
Glu	0.52	0.47–0.58	0.001	0.53	0.48–0.59	< 0.001	0.57	0.51–0.64	0.2	0.54	0.47–0.62	0.56

CTI, high-sensitive C-reactive protein-triglyceride-glucose index; WBC, White Blood Cell; LDL-C, Low-Density Lipoprotein Cholesterol; hsCRP, high-sensitivity C-Reactive Protein; TG, Triglyceride; Glu, Glucose; AUC, Area Under the Curve; CI, Confidence Interval; TCFA, thin cap fibroatheroma

## Discussion

Inflammation and metabolic dysfunction are considered essential factors in the development of various cardiovascular diseases. This study is the first to systematically investigate the relationship between the CTI index (a novel “metabolism-inflammation” composite marker) and coronary plaque characteristics in CAD patients, using multimodal imaging techniques that combine coronary angiography and OCT.

The core findings are as follows: (1) High CTI is associated with prominent high-risk/vulnerable plaque characteristics, increased incidence of macrophage aggregation, healed plaques, cholesterol crystals, multiple healed plaques, and plaque rupture, only in patients with ACS. (2) CTI is linearly negatively associated with FCT and luminal area, and linearly positively associated with lipid plaque length and the luminal area stenosis rate. (3) CTI is independently associated with vulnerable characteristics of coronary plaque, including healed plaque, cholesterol crystals, macrophage accumulation, and plaque rupture. Notably, subgroup and formal interaction analyses revealed that this independent effect was predominantly confined to patients with ACS. (4) As an univariate factor, CTI exhibits the optimal discriminative performance for vulnerable plaques compared with other inflammatory or metabolic indicators.

Hs-CRP has been established as a key risk factor for atherosclerosis and cardiovascular diseases. Investigations have demonstrated that higher hs-CRP levels are linked to larger lipid cores, more macrophage infiltration, thin-cap fibroatheromas, and a heightened risk of myocardial infarction, confirming its association with adverse plaque characteristics [21, 35, 36]. However, Mendelian randomization studies have yielded conflicting results regarding its causal role, and previous anti-inflammatory intervention trials have shown inconsistent outcomes [37–39]. These findings indicate that inflammation represents just one element of the complex pathogenesis of atherosclerosis, with hs-CRP failing to capture other key contributing factors, such as metabolic dysregulation [36].

In the field of metabolic indicators, the TyG index acts as a substitute for detecting insulin resistance and is a widely validated integrated measure of glucose and lipid metabolism. Its association with atrial stiffness and CVD events has been confirmed by multiple studies [14, 40–42]. However, the TyG index focuses solely on metabolic disorders and does not account for inflammation, which is a key driver of atherosclerotic progression. As a result, some earlier clinical studies were unable to investigate reverse causality in the application of the TyG index for these CVD patients [41, 43].

Unlike the above single-dimensional indicators, CTI integrates two core dimensions: glucose and lipid metabolism (TG, Glu) and inflammation (CRP), effectively addressing the limitations of traditional indicators. Increasing data from major prospective cohort studies like the China Health and Retirement Longitudinal Study (CHARLS) and the National Health and Nutrition Examination Survey (NHANES), confirms that a higher CTI is independently connected to an elevated risk of incident CVD, including coronary heart disease and cardiovascular mortality [24, 25, 27]. Notably, the discriminative performance of CTI has been shown to be superior to that of individual components (TyG or CRP alone), highlighting its ability to serve as a full-scale risk classification tool in clinical practice [24, 25]. However, the mechanistic basis for this association remains unclear.

Our core findings suggested that in ACS patients, increased CTI levels are closely connected with a higher incidence of several key plaque features visualized by OCT, including macrophage accumulation, cholesterol crystal deposition, and plaque rupture. Multivariate regression analysis further confirmed that this association remained independent of traditional cardiovascular risk factors such as age, gender, hypertension, and dyslipidemia. These OCT-detected vulnerability features were strongly correlated with an elevated risk of major adverse cardiovascular events (MACE) [44, 45]. These results directly demonstrate that CTI elevation is closely linked to coronary vulnerable plaque. This finding may provide a mechanistic explanation for the previously established positive correlation between CTI and increased cardiovascular event risk, as vulnerable plaques serve as the pathological basis for ACS, such as MI and unstable angina [4–7]. Thus, our data provide mechanistic support for CTI as a biological marker for predicting cardiovascular event risk, which is consistent with the conclusions of prior relevant studies [22, 24–27].

Notably, further subgroup and interaction analyses stratified by clinical presentation revealed that the above associations between CTI and vulnerable plaque phenotypes were predominantly restricted to patients with ACS, whereas no significant correlations were observed in those with stable angina pectoris (SAP). Significant effect modification by clinical presentation was formally confirmed via interaction testing, particularly for cholesterol crystals and plaque rupture. The divergent findings between ACS and SAP may be attributed to the distinct inflammatory milieu: patients with ACS exhibit acute systemic inflammation and enhanced local plaque inflammatory activity, which amplifies the impact of CTI-related metabolic and inflammatory perturbations on plaque destabilization. In contrast, SAP patients are characterized by a relatively quiescent inflammatory state, with attenuated macrophage activity and fewer high-risk plaque features, which may mask the association between CTI and coronary plaque vulnerability. Collectively, our stratified analyses reveal that the independent relationship between CTI and plaque vulnerability is primarily observed in patients with ACS.

Another important observation from this study is the significant positive association between elevated CTI and the incidence of healed plaques, particularly the presence of two or more healed plaques in ACS patients. Accumulating evidence from previous studies has shown that individuals with healed plaques frequently present with elevated high-sensitivity C-reactive protein and more severe luminal stenosis. Notably, optical coherence tomography (OCT) assessments reveal a significantly elevated incidence of plaque rupture, thin-cap fibroatheroma, and macrophage accumulation in lesions with healed plaques [46, 47]. The development of a new layered structure (healed plaque) may temporarily stabilize the plaque, but it can lead to luminal narrowing and adverse clinical outcomes [48, 49]. Nevertheless, the clinical significance of healed plaques remains controversial. While a body of evidence supports that such lesions may reflect a transient unstable state with persistent vulnerability, alternative perspectives suggest that layered structures may also represent a stabilized phase following prior subclinical plaque events.

Our results enhance this understanding by demonstrating that CTI elevation is linked to both an increased burden of vulnerable plaques and a higher prevalence of healed plaques. This suggests that patients with high CTI levels may experience a continuous cycle of plaque instability and healing, which further aggravates lumen stenosis and the risk of future heart-related incidents.

In addition to the qualitative analysis of plaque features, our study also reveals that elevated CTI is correlated with quantitative indicators of coronary artery lesions, such as increased lipid plaque length and a greater area of stenosis. These quantitative OCT findings are consistent with the results of CAG, which show that elevated CTI levels are correlated with longer lesion lengths, an increased prevalence of multi-vessel disease, and more complex lesions. Collectively, these data may provide a relatively comprehensive characterization of the correlation between CTI and CAD, from microstructural plaque vulnerability to macrostructural lesion severity.

The potential mechanisms underlying the association between CTI and coronary vulnerable plaques may be attributed to the dual role of CTI in reflecting both metabolic disorders and inflammatory responses. As a composite index integrating metabolic and inflammatory parameters (glucose, triglyceride, and hs-CRP), CTI illuminates the bidirectional crosstalk between metabolic dysfunction and inflammation in the pathogenesis and development of atherosclerosis. On one hand, metabolic abnormalities like insulin resistance and lipid disorders can promote lipid accumulation in the vascular intima, laying the foundation for plaque formation [50–52]. Triglyceride-rich lipoprotein remnants are involved in the onset and development of atherosclerotic lesions. Once retained in the subendothelial space, these particles lead to inflammation, cholesterol deposition, and macrophage foam cell formation [51]. Insulin resistance can encourage both the formation of atherosclerotic plaques and their advanced development. It likely involves systemic factors and impaired insulin signaling in intimal cells associated with atherosclerosis. On the other hand, chronic inflammatory responses can induce macrophage infiltration and activation, which leads to the degradation of the fibrous cap and subsequent plaque destabilization [53]. Consistently, a recent imaging-based case report further corroborates this notion, demonstrating that intense inflammatory burden within culprit lesions, visualized via advanced coronary imaging modalities, is strongly implicated in the onset of acute coronary events such as STEMI [54]. Inflammatory mediators released during the inflammatory response perturb insulin-mediated vasoregulation of nitric oxide and endothelin-1, exacerbating insulin resistance and forming a vicious cycle of ‘metabolic disorder → inflammatory activation → further metabolic imbalance’ [55, 56]. This reciprocal interaction perpetuates vascular endothelial injury, drives atherosclerotic plaque formation and progression, and compromises plaque stability, ultimately serving as a pivotal pathological underpinning for adverse cardiovascular events.

Another finding of the present study was that CTI demonstrated superior AUC values for identifying vulnerable plaque characteristics compared with individual inflammatory or metabolic biomarkers. The incremental discriminative value of CTI may stem from its composite imaging signatures, which integrate both inflammatory and metabolic burdens and capture more comprehensive pathological information than single conventional biomarker. Nevertheless, the overall discriminative ability of CTI for vulnerable plaques remained only moderate. Future studies incorporating additional risk factors or risk scoring systems are therefore warranted to further improve its discriminative performance.

This research demonstrates several strengths. First, it is the first investigation to explore the association between CTI and coronary plaque characteristics using OCT, providing micro-level evidence for the role of CTI in CAD. Second, the combination of CAG and OCT allows for a comprehensive assessment of both macro-level lesion severity and micro-level plaque vulnerability. Third, multivariate regression analyses were conducted to adjust for possible confounding factors, ensuring the independence of the association between CTI and plaque characteristics.

However, some limitations should be recognized. First, given the cross-sectional design of this study, temporal sequence and causality between CTI and plaque characteristics cannot be established. Longitudinal studies are required to observe whether changes in CTI over time affect plaque stability and the risk of acute cardiovascular-related incidents. Second, OCT was performed only in culprit lesions, which inherently show more vulnerable features. This limits generalizability and may exaggerate associations between CTI and plaque vulnerability. Furthermore, a considerable number of patients were excluded owing to OCT images that were unsuitable for analysis, which inevitably resulted in selection bias. Third, the results of the study, conducted at a single center, may not be widely applicable. Future large-scale multicenter studies are required to validate our results. Fourth, although CTI showed better discriminative abilities than triglyceride, glucose and hs-CRP, its ability to discriminate, as indicated by the AUC, was still moderate.

Future research should focus on the following aspects: (1) Conducting prospective cohort studies to verify whether CTI can predict the progression of vulnerable plaques and the occurrence of acute cardiovascular-related incidents. (2) Exploring the optimal cutoff value of CTI for identifying high-risk plaques to guide clinical decision-making. (3) Investigating whether targeted interventions to reduce CTI, such as lifestyle modifications or pharmacological therapy, can stabilize vulnerable plaques and improve long-term outcomes. (4) Combining CTI with other biomarkers (e.g., lipoprotein(a), high-sensitivity troponin) and imaging techniques can help establish a more comprehensive risk prediction model for vulnerable plaques.

## Conclusions

In summary, our research indicates that in patients with ACS, increased CTI levels are independently related to features of coronary vulnerable plaque assessed by OCT, including macrophage accumulation, cholesterol crystal deposition, healed plaque and plaque rupture. There is also a linear correlation between CTI and various lipid plaque-related parameters. As a single factor, CTI provides a superior discriminative value for vulnerable plaque. Given the cross-sectional design, our findings only support a significant association between CTI and plaque characteristics, rather than a causal effect. Further prospective longitudinal studies are required to verify whether CTI can predict plaque progression or events.

The authors confirm that there were no commercial or financial relationships involved in the research that could be perceived as potential conflicts of interest.

## Data Availability

The data supporting this study are available from the corresponding author upon reasonable request.

## Abbreviations

CTI: High-sensitivity C-reactive protein-triglyceride-glucose index
hs-CRP: High-sensitivity C-reactive protein
TyG: Triglyceride-glucose index
OCT: Optical coherence tomography
CAG: Coronary angiography
ACS: Acute coronary syndrome
MI: Myocardial infarction
AP: Angina pectoris
RCS: Restricted cubic splines
ROC: Receiver operating characteristics
AUC: Area under the curve
CAD: Coronary artery disease
TCFA: Thin-cap fibroatheroma
FCT: Fibrous cap thickness
LDL-C: Low-density lipoprotein cholesterol
AS: Area stenosis
WBC: White blood cell
UA: Uric acid
